# Investigation of association of genetic variant rs3918242 of matrix metalloproteinase-9 with hypertension, myocardial infarction and progression of ventricular dysfunction in Irish Caucasian patients with diabetes: a report from the STOP-HF follow-up programme

**DOI:** 10.1186/s12872-021-01860-7

**Published:** 2021-02-12

**Authors:** Chris Watson, J. Paul Spiers, Max Waterstone, Adam Russell-Hallinan, Joseph Gallagher, Kenneth McDonald, Cristin Ryan, John Gilmer, Mark Ledwidge

**Affiliations:** 1 STOP-HF Unit, St. Vincent’s University Healthcare Group, Dublin, Ireland; 2 Wellcome-Wolfson Institute for Experimental Medicine, Queen’s University , Belfast, Northern Ireland; 3 School of Medicine, University College Dublin, Dublin, Ireland; 4 Department of Pharmacology and Therapeutics, Trinity College Dublin, Dublin, Ireland; 5 School of Pharmacy and Pharmaceutical Sciences, Trinity College Dublin, Dublin, Ireland

**Keywords:** MMP-9, Diabetes, Hypertension, Myocardial infarction, Imaging, Heart failure, Prevention, Genetics, Single nucleotide polymorphism

## Abstract

**Background:**

Hypertension and/or myocardial infarction are common causes of heart failure in Type 2 diabetes. Progression to heart failure is usually preceded by ventricular dysfunction, linked to matrix metalloproteinase (MMP) mediated extracellular matrix changes. We hypothesise that the minor allele of genetic variant rs3918242 in the promoter region of the MMP-9 gene is associated with hypertension and/or myocardial infarction, with resultant progression of dysfunctional cardiac remodelling in patients with diabetes without symptomatic heart failure.

**Methods:**

We genotyped 498 diabetes patients participating in the St Vincent’s Screening TO Prevent Heart Failure (STOP-HF) follow-up programme for the rs3918242 single nucleotide polymorphism and investigated associations with the co-primary endpoints hypertension and/or myocardial infarction using a dominant model. We also evaluated resulting cardiometabolic phenotype and progression of ventricular dysfunction and cardiac structural abnormalities over a median follow-up period of 3.5 years.

**Results:**

The CT/TT genotype comprised 28.1% of the cohort and was associated with a twofold higher risk of myocardial infarction (17.9% vs 8.4%), a reduction in ejection fraction and greater left ventricular systolic dysfunction progression [adjusted OR = 2.56 (1.09, 6.01), *p* = 0.026] over a median follow-up of 3.5 years [IQR 2.6, 4.9 years]. Conversely, rs3918242 was not associated with hypertension, blood pressure, pulse pressure or left ventricular mass index at baseline or over follow up.

**Conclusions:**

Diabetes patients with the minor T allele of rs3918242 in the STOP-HF follow up programme have greater risk of myocardial infarction, lower ejection fraction and greater progression of left ventricular systolic abnormalities, a precursor to heart failure. These data may support further work on MMP-9 as a biomarker of ventricular dysfunction and the investigation of MMP-9 inhibitors for heart failure prevention in diabetes, particularly in the post-infarction setting.

*ClinicalTrials.gov Identifier*: NCT00921960

## Background

The growth of cardiovascular disease in diabetes, including hypertension, myocardial infarction and diabetes-related cardiomyopathy, is a persistent global public health problem [1, 2]. In particular, heart failure risk remains high in type 2 diabetes, despite good risk factor control [2]. The most common clinical precursors to heart failure in diabetes are hypertension and myocardial infarction, causing ventricular dysfunction and extracellular matrix (ECM) changes with diabetes-related arterial and myocardial remodelling [3–6]. The major regulators of cardiovascular ECM are matrix metalloproteinases (MMPs), in particular the gelatinases MMP-2, MMP-9 and their tissue inhibitors [7, 8]. MMP-9 is of particular importance and is a zinc dependent endopeptidase enzyme, which is expressed in a broad range of cardiovascular cell types, including cardiomyocytes, cardiac fibroblasts, vascular endothelial cells and inflammatory cells [9, 10].

Under normal physiological conditions, gene expression and protein levels of MMP-9 are low [11]. However, in pathological states involving inflammation, particularly in the setting of diabetes, MMP-9 is highly expressed and plays a central role in the turnover of ECM components such as collagen, proteoglycans and elastin [12–14]. MMP-9 has also been implicated in the activation of the pro-fibrotic cytokine TGFβ, which is a key driver of cardiac fibrosis in a range of pathological conditions [15]. In animal models of diabetic ischemia/reperfusion injury, MMP-9 expression is associated with myocardial remodelling in the infarct region as well as adverse microvascular remodelling [16, 17]. In patients, MMP-9 increase in cardiovascular disease can be driven by multiple factors, one of which is via promoter-activating single nucleotide polymorphisms (SNPs). The minor allele of the SNP 1562C > T (rs3918242, https://www.ncbi.nlm.nih.gov/snp/?term=rs3918242) near the promoter region of the MMP-9 gene has been associated with increased circulating levels of MMP-9 and with higher frequencies of vascular disease, especially in type 2 diabetes [18, 19]. Furthermore, studies have demonstrated that this promoter region  polymorphism is associated with increased risk of clinical cardiovascular events in patients with diabetes or metabolic syndrome [20]. Case control studies have suggested associations with isolated systolic hypertension, although this has not been replicated in other reports [21]. Finally, although rs3918242 is associated with coronary atherosclerosis and outcome in heart failure [22, 23], the association between rs391824 genotype, hypertension, myocardial infarction and progression of left ventricular dysfunction has not been evaluated in diabetes without heart failure.

We hypothesise that the minor allele of the MMP-9 rs3918242 SNP in patients with diabetes is associated with hypertension and/or myocardial infarction in patients without symptomatic heart failure. Furthermore, we aimed to investigate the association of rs3918242 with progression of cardiac dysfunction and remodelling over follow-up. To explore this hypothesis, we genotyped diabetes patients participating in the STOP-HF study (NCT00921960) follow-up programme for the rs3918242 SNP.

## Methods

### Patient population

The study cohort comprises Irish, Caucasian patients with diabetes and cardiovascular risk factors, but without symptomatic heart failure, from the St Vincent’s Screening TO Prevent Heart Failure (STOP-HF) follow-up programme [24]. A total of 498 patients with diabetes are included, some of whom had originally participated in the randomised, controlled prospective STOP-HF study between 2005 and 2011 [24]. Patients were referred by primary care physicians (PCPs) to the STOP-HF follow-up programme and included if they were aged over 40 years and had a history of one or more of: hypertension (medicated for ≥ 1 month); hypercholesterolemia, defined as total cholesterol (TC) greater than 5.0 mmol/L (4.5 mmol/L in high risk) and/or LDL-C 3.0 mmol/L (2.5 mmol/L in high risk) or on lipid-lowering therapy; obesity defined as body mass index (BMI) > 30 kg/m^2^; vascular disease including a history of angina or coronary artery disease (confirmed by angiography, or prior history of myocardial infarction), cerebrovascular disease, and peripheral vascular disease; diabetes mellitus; arrhythmia (mainly atrial fibrillation) requiring therapy; moderate to severe valvular disease. Excluded from the present analysis were those that refused to provide informed consent, those without a diagnosis of diabetes mellitus and those with a baseline diagnosis of heart failure.

### Study procedure

The STOP-HF follow-up programme was approved by the St Vincent’s University Hospital Ethics Committee and conformed to the principles of the Declaration of Helsinki. The STOP-HF clinic nurse enrolled consecutive patients and obtained written, informed consent. Details regarding the STOP-HF programme are described elsewhere [24]. In brief, the STOP-HF follow-up programme baseline visit included clinical, cardiometabolic, medication and Doppler-echocardiography assessment (see protocol below). Cardiometabolic assessment included evaluation of blood pressure, lipids, glucose and creatinine. Biochemical analysis included evaluation of blood B-type natriuretic peptide (BNP), (Triage, Biosite). Participating patients consented to scheduled clinic follow up at intervals of 1–3 years depending on risk. At the final STOP-HF follow up programme visit, all patients underwent repeat clinical, cardiometabolic, biochemical and Doppler Echocardiography assessment. Patients and PCPs provided information on hospital visits, new diagnoses and medication changes since the previous visit. All patients underwent phlebotomy for assessment of genomic DNA and rs3918242 gene variant genotyping from leukocytes (details below). Patients, clinic personnel, investigators and primary care physicians were not informed of rs3918242 status.

### Assessment of genomic DNA and rs3918242

Genomic DNA was extracted from venous blood (leukocytes) using a standard “spooling” method as described previously [25]. In brief, blood cells were pelleted by centrifugation (10 min, 2500 rpm), re-suspended in red cell lysis buffer (0.144 M NH_4_Cl, 1 mM NaHCO_3_) and incubated for 10 min at room temperature. Cells were pelleted again (10 min, 2500 rpm) and fresh red cell lysis buffer added. Following incubation and centrifugation the cell pellet was re-suspended in nuclei lysis buffer (10 mM Tris pH 7.6, 0.4 M NaCl, 2 mM EDTA Na_2_ pH 8.0) supplemented with proteinase K (400 μl/mL) and SDS (0.625%) prior to incubation overnight at 37 °C. Protein was precipitated by the addition of saturated NaCl and removed by centrifugation (3000 rpm, 15 min). The supernatant was reserved and re-centrifuged a total of 3 times. Absolute ethanol was then added to the clarified supernatant to “spool” out the genomic DNA and it was dissolved in TE buffer (1 mM EDTA and 10 mM Tris–HCl, pH 8.0). Patients were genotyped for the MMP-9—1562 C/T polymorphism using a restriction fragment length polymorphism (RFLP) assay. Fidelity of the RFLP assay was verified using double stranded gBlock gene fragments (IDT, Integrated DNA Technologies) covering the sequence of interest. In brief genomic DNA (10 ng) was amplified by PCR using a Hot start QPCR master mix (SpeTaqular, Rovalab, Germany) and sequence specific primers (forward: 5′ GCCTGGCACATAGTAGGCCC 3′; reverse: 5′ CTTCCTAGCCAGCCGGCATC 3′). Amplicons were digested at 37 °C for 24 h with SphI (New England Biolabs), separated by electrophoresis on 2% agarose gels stained with GelRed, and the image captured on a Fusion Fx imaging system (Vilber Lourmat, Marne-la- Vallée Cedex, France). All ambiguous readings were repeated, and 10% of the samples were randomly selected for reanalysis to confirm genotype. All genotyping was undertaken with the operator blinded to phenotype.

### Doppler-echocardiography protocol

Doppler-echocardiography was performed by an experienced Cardiac echocardiographer in the specialist STOP-HF unit. The evaluation was blinded to rs3918242 status as well as clinical and biochemical assessment (including BNP). A Philips IE33 ultrasound scanner with standard adult probe was used for data acquisition. All studies were performed in accordance with the American Society of Echocardiography recommendations as previously outlined [26]. Echocardiographic evaluation included: the functional measures ejection fraction (EF, Teicholz method), ratio of transmitral Doppler early filling velocity to tissue Doppler early diastolic mitral annular velocity at the lateral wall (E/e’), lateral e’; cardiac structural measures (Left atrial volume (LAV) and left ventricular mass (LVM, Devereux formula) indexed to indexed to body surface area (LAVI, LVMI respectively). Left ventricular dysfunction (LVD) was defined as either ejection fraction (EF) < 50% and/or left ventricular diastolic dysfunction (LVDD) with lateral E/e’ > 13 and/or lateral wall e’ < 9. LVD progression over the follow up period was defined as LV systolic dysfunction (LVSD) progression (follow up EF < 50% with a reduction of at least 5%) and LVDD progression (follow-up lateral E/e’ > 13, with an increase from baseline of > 2 and/or follow-up lateral e’ < 9 with a decrease from baseline of > 2). Progression of cardiac structural abnormalities was defined as follow up LAVI > 34 mL/m^2^ with an increase from baseline > 3.5 mL/m^2^ and/or progression of left ventricular hypertrophy (follow up LVMI > 125 g/m^2^ / > 110 g/m^2^ in males/females, with an increase of > 20 g/m^2^).

### Endpoints

The primary endpoint of this study is the association between rs3918242 genotype and hypertension and/or myocardial infarction. The secondary endpoints are the association of rs3918242 with cardiometabolic phenotype (BMI, lipids, blood pressure, glucose and creatinine) as well as Doppler-echocardiographic measures at baseline and over follow up.

As exploratory endpoints, we also report death, major adverse cardiovascular events (MACE) requiring hospitalisation, and all cause admissions. MACE included arrhythmia, transient ischemic attack (TIA), stroke, myocardial infarction, peripheral or pulmonary thrombosis/embolus, valvular heart disease or heart failure. All clinical end points were assessed by a member of the study team by reviewing combinations of the patient report and primary care physician referral letter with hospital discharge summary. Due to the low frequency of homozygotes for the T allele, all analyses were performed assuming a dominant model (CT and TT genotypes combined).

### Sample size calculation

With 498 evaluable subjects and a minor allele frequency of approximately 30% for the rs3918242 T allele, we anticipate 149 subjects with minor allele. This sample size gives 80% power, with a two-sided α = 0.05, to detect a 13% difference in hypertension and a 10% difference in myocardial infarction. For continuous secondary endpoints, this sample size also gives 80% power to detect a 0.25 standard deviation difference between groups, or a 1.9% difference in ejection fraction, a 2.5 mL/m^2^ difference in LAVI and a 0.85 difference in E/e’ and an approximate 13% difference in progression of LVD and cardiac structural abnormalities. The study is also powered to detect a difference of 1.4 kg/m^2^ in BMI, 5.5 mmHg SBP, and 3 mmHg DBP, which are considered within the bounds of clinical significance for these measures.

### Statistical analysis

All analyses were carried out on an intention-to-treat basis using R 3.6.1 (CRAN Project, 2019) statistical programming language. Analyses were performed by a statistician employed by the St Vincent’s University Hospital and the School of Medicine in University College Dublin. The analyses of the primary and secondary endpoints included all 498 patients. Demographics and clinical characteristics were summarized with descriptive statistics (counts and percentages for categorical variables, or medians and interquartile ranges for continuous variables). Non-normal variables including BNP, serum glucose and triglycerides levels were log transformed. To test for an association between cardiovascular phenotype and genotype, categorical cardiovascular and metabolic phenotypes were initially evaluated use Pearson’s chi squared test and for characteristics of interest later using unadjusted and then age and sex adjusted logistic regression analyses with rs3918242 genotype as the explanatory variable. Associations between genotype and continuous variables confirmed to be normally distributed (using Shapiro-Wilks test) were analysed using two-tailed t-tests. If continuous variables were transformable to normal, they were power-transformed and two-tailed t-tests used. If data were not transformable to normal, the non-parametric t-test equivalents (Wilcoxon signed rank and rank sum test) were used to test differences. Linear regression analyses were used to test for associations between genotype and continuous cardiovascular and metabolic phenotypes in age and sex adjusted analyses. A nominal 2-sided significance level of 0.05 was used with Bonferroni correction for multiple testing of the primary outcomes.

## Results

### Patient population

The distribution of rs3918242 genotypes was CC (358, 71.9%), CT (129, 25.9%) and TT (11, 2.2%), with genotype distribution in Hardy Weinberg equilibrium. Patients who carried the minor allele (CT/TT) accounted for 28.1% of the total cohort. This is similar to several reports on rs3918242 in other European populations, but not to that of a Chinese population with subarachnoid haemorrhage, where minor allele carriers accounted for almost half of the population (Additional file 1: Table S1). The baseline demographics and medical history of the population are described in Table 1 and blood biochemistry and Doppler echocardiography at baseline are presented in Table 2. Medication usage was similar in both genotype groups (Additional file 1: Table S2) at baseline and follow up. It was also typical of the patient population with 333 (66.9%) taking RAAS modifying therapies, 332 (66.7%) taking antiplatelet agents (predominantly aspirin) and 348 (69.9%) taking statins.

**Table 1:** Patient baseline demographics and medical history

	All (n = 498)	CC (n = 358)	CT/TT (n = 140)	*P*
Demographics				
Age, years, median [IQR]	66.2 [58.2:72.9]	66.0 [57.8:73.0]	67.3 [59.6:72.9]	ns
Male N (%)	297 (59.6%)	207 (57.8%)	90 (64.3%)	ns
BMI, median [IQR]	29 [26:33]	29 [26:33]	29 [26:33]	ns
SBP, median [IQR]	133 [122:149]	130 [119:142]	133 [122:149]	ns
DBP, median [IQR]	80 [72:86]	79 [72:86]	80 [73:87]	ns
Heart rate, median [IQR]	72 [63:80]	72 [63:81]	71 [64:80]	ns
Medical history				
Hypertension, n (%)	364 (73.1%)	263 (73.5%)	101 (72.1%)	ns
Myocardial infarction, n (%)	55 (11.0%)	30 (8.4%)	25 (17.9%)	0.002
Angina, IHD, n (%)	39 (7.89%)	27 (7.5%)	12 (8.6%)	ns
Hypercholesterolaemia, n (%)	381 (76.5%)	272 (76.0%)	109 (77.9%)	ns
Arrhythmia, n (%)	35 (7.0%)	28 (7.8%)	7 (5.0%)	ns
Atrial fibrillation, n (%)	38 (7.6%)	29 (8.1%)	9 (6.4%)	ns
Stroke/TIA, n (%)	33 (6.6%)	25 (7.0%)	8 (5.7%)	ns
Valvular heart disease, n (%)	6 (1.2%)	5 (81.4%)	1 (0.7%)	ns
Blood biochemistry				
BNP, median [IQR]	19.7 [7.8:54.6]	19.1 [7.8:51.1]	23.3 [7.9:57.6]	ns
Cholesterol, median [IQR]	4.1 [3.6:4.7]	4.1 [3.6:4.7]	4.1 [3.4:4.7]	ns
LDL, median [IQR]	2.1 [1.7:2.6]	2.1 [1.6:2.6]	2.0 [1.7:2.6]	ns
HDL, median [IQR]	1.1 [0.9:1.4]	1.1 [0.9:1.4]	1.1 [0.8:1.4]	ns
Triglycerides, median [IQR]	1.7 [1.1:2.4]	1.7 [1.1:2.4]	1.6 [1.2:2.5]	ns
Glucose, median [IQR]	8.2 [6.3:11.1]	8.2 [6.3:10.9]	8.7 [6.5:11.3]	ns
Creatinine, median [IQR]	87 [72:105]	89 [72:105]	82 [73:102]	ns

*IQR* interquartile range, *BMI* body mass index, *SBP* systolic blood pressure, *DBP* diastolic blood pressure, *IHD* ischemic heart disease, *TIA* transient ischaemic attack, *BNP* B-type natriuretic peptide, *LDL* low density lipoprotein cholesterol, *HDL* high density lipoprotein cholesterol

**Table 2:** Baseline blood biochemistry and Doppler-echocardiography

	All (n = 498)	CC (n = 358)	CT/TT (n = 140)	*P* for difference
Doppler-echocardiography				
EF, median [IQR]	66 [61:70]	66 [61:71]	64 [60:69]	0.005
E', median [IQR]	8.1 [6.5:10.0]	8.2 [6.7:10.0]	8.1 [6.4:10.0]	ns
E/E’, median [IQR]	8.4 [6.8:10.4]	8.5 [6.7:10.5]	8.4 [7.0:10.4]	ns
LAVI, median [IQR]	24.6 [20.9:30.1]	24.5 [20.6:30.1]	24.8 [21.5:30.1]	ns
LVMI, median [IQR]	92 [80:108]	92 [80:108]	93 [79:109]	ns
Baseline prevalence of LVD				
LVDD, n (%)	301 (60.4)	211 (58.9)	90 (64.3)	ns
LVSD, n (%)	24 (4.8)	15 (4.2)	9 (6.4)	ns
Any LVD, n (%)	312 (62.7)	217 (60.6)	95 (67.9)	ns
Any elevated LAVI, n (%)	97 (19.5)	75 (20.9)	22 (14.3)	ns
Any LVH, n (%)	71 (14.2)	49 (13.7)	22 (15.7)	ns

*IQR* interquartile range, *EF* ejection fraction, *LA* left atrium, *E’* tissue Doppler early diastolic mitral annular tissue velocity at the lateral wall, *E/e’* ratio of transmitral Doppler early filling velocity to tissue Doppler early diastolic mitral annular velocity, *LVMI* left ventricular mass index, *LAVI* left atrial volume index, *LVD* left ventricular dysfunction, *LVDD* left ventricular diastolic dysfunction, *LVSD* left ventricular systolic dysfunction

### Primary endpoint

The minor T allele was associated with a significantly higher baseline prevalence of myocardial infarction (MI), but not hypertension, in our diabetes population (Table 1). Using logistic regression, carriers of CT/TT genotype (CT/TT) were more than twice as likely to have suffered MI than carriers of CC genotype (CC) (OR = 2.37, [IQR 1.34, 4.21], *p* = 0.002) and, in adjusted analyses, this relationship remains significant (OR = 2.02, [IQR 1.13, 3.63], *p* = 0.018). There was no evidence of any association between the genotype and the risk of hypertension (OR = 0.94, [IQR 0.61,1.50], *p* = 0.77).

### Secondary endpoints

There were no differences in the cardiometabolic measures (SBP, DBP, heart rate, lipid, glucose, creatine and BMI measures) between both groups. While Doppler-echocardiographic analyses show no difference in the baseline prevalence of LVSD or LVDD between the genotypes, there was a small but significant reduction in left ventricular ejection fraction associated with the minor T allele (Table 2).

Furthermore, over a median follow-up of 3.5 years [IQR 2.6, 4.9 years] there was a significant difference in LVSD progression (Table 3) suggesting that diabetes patients with the minor T allele are also more at risk of progression of systolic abnormalities over the follow up period [unadjusted OR = 2.98 (1.19, 7.51), *p* = 0.015; adjusted OR = 2.56 (1.09, 6.01), *p* = 0.026]. There were no differences between the two groups in terms of LVDD, LVD or LVD and structural cardiac abnormality progression. There was also a significant difference between both groups in the proportion of patients demonstrating progression of LAVI [unadjusted OR = 1.92 (1.0, 3.70), *p* = 0.047], although this did not remain significant when adjusted for age and sex.

**Table 3:** Left ventricular dysfunction and structural cardiac abnormality progression

LVD progression	All (n = 498)	CC (n = 358)	CT/TT (n = 140)	OR	*P*
LVDD progression, n (%)	111 (22.3)	82 (22.9)	29 (20.7)	1.24 [0.77, 1.99]	ns
LVSD progression, n (%)	19 (3.8)	9 (2.5)	10 (7.1)	2.98 [1.19, 7.51]	0.015
Any LVD progression, n (%)	127 (25.5)	89 (24.9)	38 (27.1)	1.13 [0.72, 1.75]	ns
LAVI progression	41 (8.2)	24 (6.7)	17 (12.1)	1.92 [1.0, 3.70]	0.047
LVH progression, n (%)	33 (6.6)	24 (6.7)	9 (6.4)	0.96 [0.43, 2.11]	ns
Any LVD or SCA progression, n (%)	166 (33.3)	114 (31.8)	52 (37.1)	1.40 [0.91, 2.14]	ns

*LVD* left ventricular dysfunction, *LVDD* left ventricular diastolic dysfunction, *LVSD* left ventricular systolic dysfunction, *LVH* left ventricular hypertrophy, *LAVI* left atrial volume index, *SCA* structural cardiac abnormality

### Exploratory endpoints

There were no differences between the groups in terms of the following clinical event endpoints: death and all-cause emergency hospitalization (CC n = 88, 24.6%, vs. CT/TT n = 36, 25.7%, OR = 1.06 [0.68, 1.66], p = NS); death or MACE or LVD progression (CC n = 104, 29.1%, vs. CT/TT n = 50, 35.7%, OR = 1.36 [0.89, 2.05], p = NS); death or MACE or LVD or structural cardiac abnormality progression (CC n = 114, 31.8%, vs. CT/TT n = 52, 37.1%, OR = 1.24 [0.82, 1.87], p = NS).

Although myocardial infarction occurred in only 11% of the total population, the majority of patients with LVSD at baseline (54%) or with LVSD progression (58%) had a history of MI. Due to the strong association between the minor T allele, myocardial infarction and reduced LVEF in our study, we carried out a post-hoc analysis of patients with a history of myocardial infarction to see if severity of cardiac damage, denoted by reduced LVEF, was greater in this subset. We compared clinical characteristics, medications and Doppler echocardiography of the patients with myocardial infarction according to CC or CT/TT genotype (Additional file 1: Tables S3–S5). Carriers of the minor allele had significantly reduced EF and the magnitude of the difference in EF was greater than the wider study population, as was baseline LVSD (n = 10, 32% vs. n = 4, 16%) and progression of LVSD (n = 8, 26% vs. n = 4, 16%), although there was insufficient power to demonstrate statistical significance. As with the overall cohort, there were no baseline differences in terms of other clinical characteristics, blood biochemistry, medications and other measures of cardiovascular remodelling and diastolic dysfunction (e.g. LAVI, LVMI, e’, E\e’).

## Discussion

This study builds on a growing body of evidence that MMP-9 may contribute to cardiovascular disease in people with diabetes. Amongst diabetes patients without symptomatic heart failure carrying the minor (T) allele of rs3918242 we observed a twofold risk of prior myocardial infarction, but no associated risk of hypertension. We also found significantly lower ejection fraction at baseline in CT/TT genotyped patients and greater progression of left ventricular systolic dysfunction over a median 3.5 year follow up period, suggesting higher risk of the future development of HF. Consistent with these observations, in propensity-matched, sub-group analyses of patients with a history of myocardial infarction, CT/TT genotype patients had significantly reduced ejection fraction compared to CC controls, with greater reduction in ejection fraction compared to T allele patients in the overall cohort. As MMP-9 is an inducible gelatinase and rs3918242 C > T in the promoter region of the gene is known to augment expression of MMP-9, these data may support the use of MMP-9 as a biomarker of ventricular dysfunction and the investigation of MMP-9 inhibitors for HF prevention in diabetes, particularly in the post-infarction setting.

The observational nature of this study means that causality between rs3918242, myocardial infarction and ejection fraction cannot be definitively established. In addition, the population is exclusively Irish Caucasian, and our results may not hold in populations with different ethnicities, where minor allele frequencies differ. The work is also limited by relatively low patient number and potentially biased by the absence of a matched non-diabetic cohort to investigate whether this relationship applies only in the context of diabetes. Furthermore, while the minor T allele of rs3918242 is associated with coronary artery and cerebrovascular diseases in large clinical studies and a recent meta-analysis, [20, 27, 28] it has not emerged in GWAS of vascular diseases to date. However, in order to reduce the likelihood of false positive results due to multiple testing, current GWAS require p-values in the order of 10^–8^ and consequently identify only a minority of gene variants associated with disease risk. Moreover, GWAS may lack deep phenotypic characterisation and many of the variant candidates emerging from GWAS to date have uncertain functional significance [29]. A pragmatic approach to these challenges could be the integration of larger GWAS data with laboratory or clinical studies, particularly with deep phenotyping such as ours. Also, the use of gene or pathway-based association tests to explore the biological plausibility and clinical relevance of potential gene variants, can also investigate gene–environment interactions that are thought to explain some of the missing heritability from GWAS. It is noteworthy, in this regard, that several researchers have identified an interaction between the risk T allele of rs3918242 and diabetes and other features of metabolic syndrome [18, 19, 30].

The main strength of this study is the detailed clinical, biochemical and deep-phenotyping with sequential Doppler echocardiography to highlight the association of myocardial infarction, asymptomatic left ventricular dysfunction and progression of left ventricular systolic dysfunction amongst carriers of the rs3918242 T allele. The data may be important in the context of concerns about the burden of heart failure in diabetes and support the potential of MMP-9 as a diagnostic marker and/or therapeutic target post-MI in order to prevent heart failure in this setting. Our study is also consistent with a large population based study of coronary artery disease patients, which showed that the rs3918242 T-allele associated with the risk of clinical events only in patients with metabolic syndrome [21]. Chronic low grade inflammation in the metabolic syndrome and diabetes can lead to alterations in extracellular matrix turnover and adverse changes in myocardial connective tissue with collagen accumulation and fibrosis [3–6]. The present report builds on other studies, which show that the minor allele of rs3918242, as a promoter region SNP of the MMP-9 gene, increases circulating levels of MMP-9 and is associated with vascular disease in type 2 diabetes mellitus [18–20]. Aortic and coronary arteries of patients with diabetes taken at autopsy had higher expression of MMP-9 compared to non-diabetes patients and these levels were correlated with HbA1c as well as apoptosis [31]. Our study is also consistent with the adverse phenotype and outcome associated with rs3918242 in later stage, symptomatic HFrEF [22, 32, 33].

In contrast, we did not find any association of rs3918242 with baseline prevalence of hypertension, blood pressure and pulse pressure. This is unlike previous studies showing MMP-9 is associated with arterial stiffness in patients with diabetes [18]. Similarly, in a cohort of asymptomatic hypertensive patients, rs3918242 is associated with higher plasma MMP-9 and evidence of increased hypertension and vascular stiffness, measured by pulse wave velocity [19]. In an elderly cohort, the minor allele was associated with risk of isolated systolic hypertension [21]. We cannot exclude differences in vascular function or hypertension using more precise measures or larger populations. Similarly, we did not observe similar associations between genotype and LVDD progression, baseline measures of LVDD (E/e’, e’) and changes in left ventricular mass, features associated with hypertension and heart failure with preserved ejection fraction. A similar observation has been made in relation to left ventricular mass changes in the post-infarction setting [34]. However, the unadjusted difference in LAVI progression, a well-established continuous marker of LVDD, may suggest that associations between re3918242 and LVDD requires further evaluation in asymptomatic, at-risk patients with diabetes.

Alternatively, the present study may support a hypothesis that over-expression of MMP-9 is most associated with the evolution of, and cardiac response to, myocardial infarction in diabetes. The magnitude of EF difference at baseline, while significant, was considerably smaller in the total population rather than the prior MI subset and the majority of patients that demonstrated LVSD progression over time had prior MI. This relationship may be complex, involve the innate immune response and have important temporal features. For example, in accordance with our study, maximal plasma MMP-9, seen early in the first 12 h post-MI, is independently related to lower EF and increased circulating leukocytes [35]. On one hand, platelet activation, as occurs in myocardial infarction, results in mutual platelet and monocyte co-stimulation, extracellular matrix metalloproteinase inducer expression and secretion of monocyte MMP-9 [36]. The rs3918242 minor allele is associated with an exaggerated plasma MMP-9 response in coronary artery disease patients and, in turn, elevated plasma MMP-9 independently predicts cardiovascular mortality [27] as well as severity of MI [37]. On the other hand, animal data show that cardiac macrophage overexpression of MMP-9 is associated with preservation of ventricular function and wound healing post-MI [38]. Similarly, later plateau levels of circulating MMP-9, in contrast to earlier levels post-MI, are positively associated with preservation of LV function [35], underlining the short-term, temporal nature of adverse MMP-9 responses.

Future avenues of research could build on these data with a view to individualizing care. For example, excess MMP-9 activity associated with the minor allele of rs3918242 may be a therapeutic target in post-MI diabetes patients. To date, MMP inhibitors have produced mixed results when evaluated clinically in cardiovascular disease patients including post-MI, which may relate to temporal issues described above, as well as a lack of selectivity of inhibitors for pathophysiological MMP isoforms [39]. There may be blunting of tissue specific protective roles of MMPs in the disease setting [38]. However, intriguing data from the TIPTOP study indicates a beneficial effect on vascular and left ventricular remodelling when doxycycline, an inhibitor of MMP, is used early in patients with acute MI [40, 41], suggesting options for further research in diabetes patients carrying the minor allele of rs3918242.

## Conclusions

Diabetes patients with the minor T allele of rs3918242 in the STOP-HF follow up programme have greater prevalence of myocardial infarction, reduced ejection fraction at baseline and greater progression of left ventricular systolic dysfunction over a median 3.5-year follow-up period. The reduced ejection fraction is lower in subsets of post-myocardial infarction patients with the CT/TT genotype. In contrast, we did not find any association of rs3918242 with baseline prevalence of hypertension, blood pressure and pulse pressure. These data may support further work on MMP-9 as a biomarker of ventricular dysfunction and the investigation of MMP-9 inhibitors for HF prevention in diabetes, particularly in the post-infarction setting.

## Supplementary Information

**Additional file 1**. Supplementary Data File.

## Data Availability

The datasets generated and analysed during the current study are not publicly available due to the fact that participants in the study have not consented to publication of individual, patient-level data, but are available from the corresponding author on reasonable request.

## Abbreviations

STOP-HF: St Vincent’s Screening TO Prevent Heart Failure
MMP: Matrix metalloproteinase
OR: Odds ratio
IQR: Interquartile range
ECM: Extracellular matrix
TGFβ: Transforming growth factor β
SNP: Single nucleotide polymorphisms
Mmol: Millimolar
BMI: Body mass index
DNA: Deoxyribonucleic acid
PCP: Primary care physician
BNP: B-type natriuretic peptide
LVD: Left ventricular dysfunction,
LVSD: Left ventricular systolic dysfunction
LVDD: Left ventricular diastolic dysfunction
LAVI: Left atrial volume Indexed to body surface area
LVMI: Left ventricular mass Indexed to body surface area
RFLP: Restriction fragment length polymorphism
E/e’: Ratio of transmitral Doppler early filling velocity to tissue Doppler early diastolic mitral annular velocity
EF: Ejection fraction
MACE: Major adverse cardiovascular event
TIA: Transient ischemic attack
SBP: Systolic blood pressure
DBP: Diastolic blood pressure
RAAS: Renin angiotensin aldosterone modifying therapies
MI: Myocardial infarction
IHD: Ischemic heart disease
LDL: Low density lipoprotein cholesterol
HDL: High density lipoprotein cholesterol
SCA: Structural cardiac abnormality (elevated LAVI or LVMI)
