# Supplementary material for: Investigation of association of genetic variant rs3918242 of matrix metalloproteinase-9 with hypertension, myocardial infarction and progression of ventricular dysfunction in Irish Caucasian patients with diabetes: a report from the STOP-HF follow-up programme
Source: BMC Cardiovasc Disord. 2021 Feb 12;21:87. doi: 10.1186/s12872-021-01860-7 (PMC7879511; doi:10.1186/s12872-021-01860-7)
Supplement: Supplementary file 1 — Additional file 1. Supplementary Data File. [file 12872_2021_1860_MOESM1_ESM.docx]

| \| Report \| **Disease assessment of rs3918242** \| **Patient Cohort** \| **CC** \| **CT** \| **TT** \| **CT+TT** \| \| --- \| --- \| --- \| --- \| --- \| --- \| --- \| \| Current study \| Diabetes \| Irish (Caucasian) (n=498) \| 71.9 \| 25.9 \| 2.2 \| 28.1 \| \| PMID:32695863 \| Primary open-angle glaucoma, essential hypertension and peptic ulcer (DM was not assessed) \| Russian (n=536) \| 71.8 \| 24.8 \| 3.4 \| 28.2 \| \| PMID:28390432 \| Coronary heart disease (DM was an exclusion criteria for this study) \| Chinese (n=264) \| 71.3 \| 26 \| 2.7 \| 28.7 \| \| PMID:29763368 \| Subarachnoid hemorrhage patients (18.7% DM) \| Chinese (n=460) \| 51.3 \| 39.3 \| 9.4 \| 48.7 \| \| PMID: 21455563 \| Systemic Sclerosis (DM was not assessed) \| Italian (Caucasian) (n=228) \| 76.3 \| 22.81 \| 0.88 \| 23.69 \| \| PMID:30257242 \| Ischaemic Stroke (DM in 20.17% of cohort) \| Southern Chinese (n=1274) \| 78.7 \| 18.9 \| 2.4 \| 21.3 \| \| PMID: 17581602 \| Hypertension (DM was not assessed) \| Caucasian (n=215) \| 68 \| 29 \| 3 \| 32 \| \| PMID:25191702 \| Coronary Artery disease with metabolic sydrome (52% T2DM) \| ASCET study: 97% of Western European descent (n=244) \| 74.6 \| 24.6 \| 0.8 \| 25.4 \| \|  \| Coronary Artery disease without metabolic sydrome (10% T2DM) \| ASCET study: 97% of Western European descent (n=751) \| 76.3 \| 22 \| 1.7 \| 23.7 \|   **Table S1. Allele frequencies**  **Table S2. Medication use total population** | | | | |
| --- | --- | --- | --- | --- | --- | --- | --- | --- | --- | --- | --- | --- | --- | --- | --- | --- | --- | --- | --- | --- | --- | --- | --- | --- | --- | --- | --- | --- | --- | --- | --- | --- | --- | --- | --- | --- | --- | --- | --- | --- | --- | --- | --- | --- | --- | --- | --- | --- | --- | --- | --- | --- | --- | --- | --- | --- | --- | --- | --- | --- | --- | --- | --- | --- | --- | --- | --- | --- | --- | --- | --- | --- | --- | --- |
| N(%) | All (n=498) | CC (n=358) | CT/TT (n=140) | P |
| **Baseline Medications** |  |  |  |  |
| Any RAAS, n(%) | 333 (66.9%) | 240 (67.1%) | 93 (66.2%) | ns |
| ACEI, n(%) | 207 (41.5%) | 152 (42.5%) | 55 (39.2%) | ns |
| ARB, n(%) | 152 (30.6%) | 109 (30.4%) | 44 (31.1%) | ns |
| Aspirin, n(%) | 332 (66.7%) | 232 (64.9%) | 99 (70.9%) | ns |
| Diuretic, n(%) | 155 (31.2%) | 114 (31.8%) | 42 (29.7%) | ns |
| Alpha-blocker, n(%) | 48 (9.7%) | 32 (8.8%) | 17 (12.2%) | ns |
| Beta-blocker, n(%) | 147 (29.6%) | 101 (28.2%) | 46 (33.1%) | ns |
| CCB, n(%) | 150 (30.2%) | 105 (29.3%) | 45 (32.4%) | ns |
| OAD, n(%) | 353 (70.9%) | 244 (68.2%) | 109 (77.9%) | ns |
| Insulin, n(%) | 58 (11.6%) | 44 (12.2%) | 14 (10.0%) | ns |
| Statin, n (%) | 349 (70.0%) | 245 (68.5%) | 103 (73.6%) | ns |
|  |  |  |  |  |
| **Follow-up Medications** |  |  |  |  |
| Any RAAS, n(%) | 357 (71.7%) | 252 (70.4%) | 105 (75.0%) | ns |
| ACEI, n(%) | 220 (44.1%) | 158 (44.1%) | 61 (43.9%) | ns |
| ARB, n(%) | 154 (31.0%) | 107 (29.9%) | 47 (33.8%) | ns |
| Aspirin, n(%) | 337 (67.6%) | 236 (66.0%) | 100 (71.6%) | ns |
| Diuretic, n(%) | 185 (37.2%) | 136 (38.1%) | 49 (35.1%) | ns |
| Alpha-blocker, n(%) | 80 (16.0%) | 51 (14.2%) | 28 (20.3%) | ns |
| Beta-blocker, n(%) | 180 (36.1%) | 125 (34.8%) | 55 (39.2%) | ns |
| CCB, n(%) | 182 (36.5%) | 130 (36.2%) | 52 (37.2%) | ns |
| OAD, n(%) | 364 (36.5%) | 256 (36.2%) | 108 (37.2%) | ns |
| Insulin, n(%) | 58 (11.6%) | 44 (12.2%) | 14 (10.0%) | ns |
| Statin, n (%) | 379 (76.2%) | 273 (76.2%) | 107 (76.4%) | ns |

Abbreviations: RAAS, renin angiotensin aldosterone system modifying therapy; ACEI, ACE inhibitor; ARB, angiotensin receptor blocker; CCB, calcium channel blockers; OAD, oral anti-diabetic therapy.

| **Table S3. Patient baseline demographics, clinical history and blood biochemistry in patients with a baseline history of myocardial infarction.** | | | | |
| --- | --- | --- | --- | --- |
| Median[IQR] | All (n=56) | CC (n=31) | CT/TT (n=25) | p |
| Age, years, median [IQR] | 68.9 [64.7:73.7] | 68.4 [64.6:71.9] | 69 [65.8:74.7] | ns |
| Male, n (%) | 44 (78.6%) | 24 (77.4%) | 20 (80%) | ns |
| BMI, median [IQR] | 29.4 [26.7:32.6] | 29.7 [27:32.7] | 29.2 [26.3:32.4] | ns |
| SBP, median [IQR] | 136 [121:150] | 135 [118:147] | 138 [124:152] | ns |
| DBP, median [IQR] | 76.5 [69.8:83] | 76 [71.5:81] | 78 [69:88] | ns |
| Heart Rate, median [IQR] | 66 [59:75.5] | 62.5 [57.2:71] | 70 [63:77] | ns |
| **Medical Profile/History** |  |  |  |  |
| Hyptertension, n(%) | 41 (73.2%) | 25 (80.6%) | 16 (64%) | ns |
| Arrhythmia, n(%) | 5 (8.9%) | 3 (9.7%) | 2 (8%) | ns |
| Atrial Fibrillation, n(%) | 13 (23.2%) | 5 (16.1%) | 8 (32%) | ns |
| Stroke/TIA, n (%) | 6 (10.7%) | 4 (12.9%) | 2 (8%) | ns |
| **Bloods** |  |  |  |  |
| BNP, median [IQR] | 60.8 [22.9:102] | 56.2 [21.1:95.5] | 62.7 [37:106] | ns |
| Cholesterol, median [IQR] | 3.9 [3.4:4.4] | 3.8 [3.3:4.4] | 3.9 [3.5:4.5] | ns |
| LDL, median [IQR] | 1.8 [1.5:2.2] | 2 [1.6:2.1] | 1.7 [1.4:2.4] | ns |
| HDL, median [IQR] | 0.99 [0.81:1.2] | 1 [0.92:1.2] | 0.87 [0.76:1.2] | ns |
| Triglycerides, median [IQR] | 1.7 [1.2:2.7] | 1.7 [1.2:2.6] | 1.6 [1.2:3] | ns |
| Glucose, median [IQR] | 7.4 [5.8:10.6] | 6.8 [5.9:10.9] | 7.8 [5.8:9] | ns |
| Stroke/TIA, n (%) | 6 (10.7%) | 4 (12.9%) | 2 (8%) | ns |

Abbreviations: IQR, interquartile range; BMI, body mass index; SBP, systolic blood pressure; DBP, diastolic blood pressure; TIA, transient ischaemic attack; BNP, B-type natriuretic peptide; LDL low density lipoprotein cholesterol; HDL, high density lipoprotein cholesterol.

| **Table S4. Medication use in patients with a baseline history of myocardial infarction** | | | | |
| --- | --- | --- | --- | --- |
| N(%) | All (n=56) | CC (n=31) | CT/TT (n=25) | Chisq./Fisher P |
| Baseline Meds |  |  |  |  |
| Any RAAS, n(%) | 41 (73.2%) | 24 (77.4%) | 17 (68%) | ns |
| ACEI, n(%) | 26 (46.4%) | 15 (48.4%) | 11 (44%) | ns |
| ARB, n(%) | 18 (32.1%) | 12 (38.7%) | 6 (24%) | ns |
| Aspirin, n(%) | 47 (83.9%) | 27 (87.1%) | 20 (80%) | ns |
| Diuretic, n(%) | 19 (33.9%) | 8 (25.8%) | 11 (44%) | ns |
| Alpha-blocker, n(%) | 10 (17.9%) | 5 (16.1%) | 5 (20%) | ns |
| Beta-blocker, n(%) | 37 (66.1%) | 22 (71%) | 15 (60%) | ns |
| CCB, n(%) | 21 (37.5%) | 12 (38.7%) | 9 (36%) | ns |
| OAD/Insulin | 43 (76.8%) | 25 (80.1%) | 18 (72%) | ns |
| Statin, n (%) | 51 (91.1%) | 28 (90.3%) | 23 (92%) | ns |
|  |  |  |  |  |
| Follow-up Meds |  |  |  |  |
| Any RAAS, n(%) | 44 (78.6%) | 24 (77.4%) | 20 (80%) | ns |
| ACEI, n(%) | 30 (53.6%) | 17 (54.8%) | 13 (52%) | ns |
| ARB, n(%) | 18 (32.1%) | 11 (35.5%) | 7 (28%) | ns |
| Aspirin, n(%) | 47 (83.9%) | 28 (90.3%) | 19 (76%) | ns |
| Diuretic, n(%) | 25 (44.6%) | 12 (38.7%) | 13 (52%) | ns |
| Alpha-blocker, n(%) | 16 (28.6%) | 9 (29%) | 7 (28%) | ns |
| Beta-blocker, n(%) | 46 (82.1%) | 26 (83.9%) | 20 (80%) | ns |
| CCB, n(%) | 24 (42.9%) | 14 (45.2%) | 10 (40%) | ns |
| OAD/Insulin | 41 (73.2%) | 24 (77.4%) | 17 (68.0%) | ns |
| Statin, n (%) | 54 (96.4%) | 30 (96.8%) | 24 (96%) | ns |

Abbreviations: RAAS, renin angiotensin aldosterone system modifying therapy; ACEI, ACE inhibitor; ARB, angiotensin receptor blocker; CCB, calcium channel blockers; OAD, oral anti-diabetic therapy.

| **Table S5. Doppler Echocardiography in patients with a baseline history of myocardial infarction.** | | | | |
| --- | --- | --- | --- | --- |
| Median[IQR] | All (n=56) | CC (n=31) | CT/TT (n=25) | Wilcoxon p |
|  |  |  |  |  |
| EF, median [IQR] | 56.5 [49.8:63.5] | 60 [55:67.8] | 50 [45:59] | 0.003 |
| E', median [IQR] | 7.9 [6.4:10] | 8 [7.2:9.8] | 6.8 [5.1:10.8] | ns |
| E/E’, median [IQR] | 9.3 [7.5:11.8] | 8.6 [7.5:10.5] | 10.6 [7.5:12.1] | ns |
| LAVI, median [IQR] | 30.7 [25.8:34.8] | 30.5 [26.2:35.2] | 30.8 [25.5:34.7] | ns |
| LVMI, median [IQR] | 107 [92:125] | 105 [92.3:115] | 115 [90.7:142] | ns |
|  |  |  |  |  |

Abbreviations: IQR, interquartile range; EF, ejection fraction; LA, left atrium; E’, tissue Doppler early diastolic mitral annular tissue velocity at the lateral wall; E/e’, ratio of transmitral Doppler early filling velocity to tissue Doppler early diastolic mitral annular velocity; LVMI, left ventricular mass index; LAVI, left atrial volume index; LVD, left ventricular dysfunction; LVDD left ventricular diastolic dysfunction; LVSD, left ventricular systolic dysfunction
